# *Eggerthella lenta* down regulated flavone and flavonol biosynthesis promoted Kawasaki disease

**DOI:** 10.1080/21505594.2025.2512401

**Published:** 2025-05-31

**Authors:** Yao-Tsung Yeh, Kuang-Den Chen, Cheng-Hsieh Huang, Jia-Rong Tsai, Ho-Chang Kuo

**Affiliations:** a Aging and Diseases Prevention Research Center, Fooyin University, Kaohsiung, Taiwan; b Department of Medical Laboratory Science and Biotechnology, Fooyin University, Kaohsiung, Taiwan; c Institute for Translational Research in Biomedicine, Kaohsiung Chang Gung Memorial Hospital, Kaohsiung, Taiwan; d Kawasaki Disease Center and Department of Pediatrics, Kaohsiung Chang Gung Memorial Hospital and College of Medicine, Chang Gung University, Kaohsiung, Taiwan; e Ph.D. Program in Environmental and Occupational Medicine, College of Medicine, Kaohsiung Medical University and National Health Research Institutes, Kaohsiung, Taiwan; f Department of Respiratory Therapy, Kaohsiung Chang Gung Memorial Hospital, Kaohsiung, Taiwan

**Keywords:** Kawasaki disease, *Bacteroides ovatus*, *Eggerthella lenta*, flavone, flavonol

## Abstract

Kawasaki Disease (KD) is a multisystemic vasculitis of unknown aetiology in children. The incidence of KD varies by geographic area and correlates with differences in gut microbiota patterns, with the highest incidence in Asian. This study aimed to investigate alterations in faecal microbiota and assess their relationship with systemic inflammation in KD patients. A total of 59 patients and 55 matched controls were included. Fecal samples were collected at the onset of KD. The V3/V4 regions of 16S rDNA were sequenced using the MiSeq platform. PICRUSt 2 was used to analyse the potential functional pathways involved in gut dysbiosis. Alpha (*p* < 0.042) and beta (*p* < 0.001) diversity in KD were significantly decreased when compared to the control group. After multivariate regression, among the seven critical microbes, increased *Eggerthella lenta* (*p* = 0.016) and decreased Bacteroides ovatus (*p* = 0.014) could also predict KD risk using receiver operating characteristic curve (ROC) analysis (*Eggerthella lenta*: area under the ROC curve, AUC = 0.841, odds ratio = 23.956; Bacteroides ovatus: AUC = 0.816, odds ratio = 31.365). Notably, *Bacteroides ovatus* was positively correlated with blood segment cells (*p* = 0.006), but negatively correlated with blood lymphocytes (*p* = 0.013). After multivariate regression, flavone and flavonol biosynthesis decreased in children with KD (*p* < 0.001). Our results indicated that both *Bacteroides ovatus* and *Eggerthella lenta* may deregulate flavone and flavonol biosynthesis, consequently modulating immune cells and potentially triggering KD. This study suggests that alterations in the gut microbiota are closely associated with immune responses and provides a new perspective on the aetiology, pathogenesis, and treatment of KD.

## GRAPHICAL ABSTRACT

**Figure:**
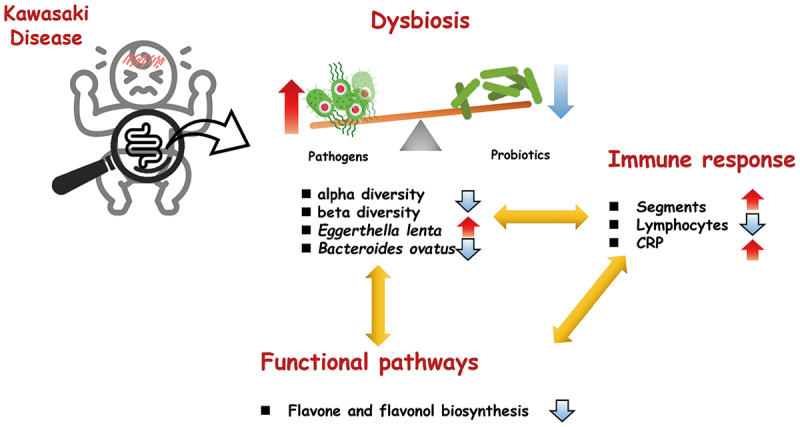
Microbial dysbiosis and immune dysregulation in Kawasaki disease: linking functional pathways to disease Pathogenesis. gut microbiota changed and decreased alpha diversity in turn caused gut dysbiosis in Kawasaki disease. The increased incidence of Kawasaki disease may be caused by an increase in *Eggerthella lenta* and a concomitant decrease in *bacteroides ovatus*, affecting the pathway of flavone and flavonol biosynthesis in patients with Kawasaki disease.

## Introduction

Kawasaki disease (KD) is an acute febrile illness characterized by multisystemic vasculitis that primarily affects small- and medium-sized muscular arteries, particularly the coronary arteries [1]. Recently, KD has been considered the leading cause of acquired heart disease in developed countries, including Taiwan and Japan. Moreover, KD is a risk factor for cardiac disease and other pathological conditions as affected children grow older [2]. However, the aetiology of KD remains poorly understood. Disturbances in the gut microbiota have been shown to induce several diseases, including inflammatory bowel, allergic, and autoimmune diseases [3,4]. Mounting evidence indicates that changes in gut microbiota composition play a significant role in the pathogenesis of KD. Specifically, there was an observed increase in the proportion of potentially harmful gram-negative bacteria that produce HSP60. These bacteria included *Neisseria, Acinetobacter, Enterobacter*, and *Veillonella*. Additionally, there is an elevated presence of gram-positive cocci with superantigenic properties, such as Streptococcus and Staphylococcus, in children with KD compared to that in healthy controls. These findings highlight the potential role of gut microbiota dysbiosis in KD development and progression [5,6]. However, the specific gut bacteria which might involve in KD and potential mechanism still unclear.

*Eggerthella lenta* has been reported to inhibit the Th17 master transcription factor, Rorγt) [7,8] and emerging evidence suggests that *E. lenta* is enriched in patients with multiple autoimmune diseases [9,10]. KD is associated with higher levels of interleukin (IL)-17A and IL-6, a cytokine profile similar to that observed in other autoimmune diseases. Intravenous immunoglobulin (IVIG) therapy increased the expression of Treg-related FoxP3. IVIG resistance is associated with high levels of IL-10 and IL-17A. These findings provide further evidence that KD is an autoimmune disease [11]. Moreover, *Bacteroides ovatus* has been studied in relation to intestinal microbiota alterations, epithelial permeability, cytokine expression, and autoimmune and innate immune responses. *B. fragilis* and *B. ovatus* have been shown to assist in maintaining intestinal equilibrium by preserving the diversity of the gut microbiota and alleviating LPS-induced inflammation, either by modulating cytokine production or restoring the Treg/Th-17 balance [12]. Th17 imbalance is an important factor in KD development, but the relationship between the gut microbiota and KD is still unclear. In this study, the increased incidence of KD may have been caused by an increase in *Eggerthella lenta* and a concomitant decrease in *Bacteroides ovatus*, affecting the pathway of flavone and flavonol biosynthesis in patients with KD was first reported.

## Methods

### Human sample collection

Fecal samples and blood tests were obtained from participants following a protocol approved by the Institutional Review Board of Chang Gung Medical Foundation (IRB codes: 102-1015A3 and 201700509B0). Written informed consent was obtained from the parents or guardians of all the children included in this study. Declaration of Helsinki was followed. Laboratory features obtained from routine blood tests included demographic characteristics (age and sex), complete blood count with differential count (CBC/DC), C-reactive protein (CRP), and alanine aminotransferase (ALT). Laboratory blood test data for patients with KD were obtained within 24 h of the first intravenous immunoglobulin (IVIG) treatment. All KD patients included in the study met the diagnostic criteria of the American Heart Association (AHA) and received IVIG at 2 g/kg body weight for 10–12 hours [13].

### Metagenomic sequencing and 16S rRNA gene pyrosequencing of human stool samples

Faecal samples were suspended in an equal volume of stool DNA stabilizer buffer (Invitrogen Molecular) and stored at −80°C until use. After thawing, 2 ml of the faecal suspension was processed to remove the stabilizer buffer by centrifugation at 13,200 rpm for 1 min. Stool DNA extraction was performed according to the manufacturer’s protocol using a QiAamp DNA Stool Mini Kit (Qiagen). The extracted DNA was stored in 1X ATE buffer (10 mm Tris-Cl pH 8.3, 0.1 mm EDTA, and 0.04% NaN3) at −20°C for subsequent 16S rRNA-targeted sequencing.

Fecal samples from the two groups were characterized for microbiome composition using 16S rRNA amplicon sequencing. Samples from participants undergoing antibiotic treatment were excluded from this study. 16S rRNA sequencing was conducted on the MiSeq platform following the Illumina protocol. PCR was performed using the forward primer 5′- TCG TCG GCA GCG TCA GAT GTG TAT AAG AGA CAG CCT ACG GGN GGC WGC AG-3′ and reverse primer 5′- GTC TCG TGG GCT CGG AGA TGT GTA TAA GAG ACA GGA CTA CHV GGG TAT CTA ATC C-3′ for the V3–V4 region of the 16S rRNA gene. Amplicons generated from each sample (~330 bp) were purified using AMPure XP magnetic beads (Beckman Coulter). DNA was quantified using a Qubit dsDNA assay kit (Invitrogen) and the samples were stored at 4°C. The pooled amplicon library was sequenced using the MiSeq Reagent Kit v3 (600 cycles) on a MiSeq sequencer (Illumina) to generate paired-end reads of 2 × 300 bp. After demultiplexing the 16S sequence reads based on sample-specific indices, primer sequences were trimmed using Cutadapt v.1.15. The trimmed reads were then processed using the DADA2 R package v.1.18.0 to construct amplicon sequence variants (ASVs). This was achieved using the filterAndTrim function with the standard parameters (maxN = 0, truncQ = 2, and maxEE = 2). Possible chimeric reads were removed using the remote Bimera Denovo function of DADA2. Taxonomic assignment of each amplicon sequence variant (ASV) was performed by similarity searching against the Greengene v13.8 database with a threshold of 97% similarity. The rarefied feature table was then converted into the linear discriminant analysis effect size (LEfSe) format using tools available on Galaxy Hutlab (http://huttenhower.sph.harvard.edu/galaxy/) and MicrobiomeAnalyst (https://www.microbiomeanalyst.ca/MicrobiomeAnalyst/home.xhtml). Alpha-diversity was assessed using the Shannon, Chao1, Abundance-based Coverage Estimator (ACE), Simpson and Fisher diversity indices. The Shannon index considers both the richness (number of species) and/or evenness (distribution of species) within each sample. Microbial communities from both the KD group and the control group were analysed for alpha-diversity. All calculations and visualizations of the Alpha-diversity indices were performed using MicrobiomeAnalyst package (https://github.com/xia-lab/MicrobiomeAnalystR). Statistical significance between the two groups was evaluated using the Wilcoxon rank-sum test, with a p-value of less than 0.05 considered significant. The diversity values were visualized as a scatter plot, where each data point represents an individual sample, and group medians are indicated. Beta-diversity was analysed using the Jaccard Index, a metric that quantifies dissimilarity based on the presence or absence of taxa. The similarity of Beta-diversity was assessed using two different statistical tests: Permutational Multivariate Analysis of Variance (PERMANOVA) and Analysis of Similarities (ANOSIM). To visualize these differences, Principal Coordinates Analysis (PCoA) was performed, generating two-dimensional plots to display clustering patterns between the KD group and the control group. Beta-diversity calculations and visualizations were also conducted using the MicrobiomeAnalyst package. Statistical significance of the differences in beta-diversity between the groups was tested using PERMANOVA (Permutational Multivariate Analysis of Variance), with a *p*-value of less than 0.001 considered significant.

For the microbial composition analysis, the data were normalized using total sum scaling, which involved dividing each feature count by the total library size. After converting the rarefied feature table into the LEfSe format using Galaxy Hutlab and MicrobiomeAnalyst, a Kruskal-Wallis test (which in the two-group case simplifies to a Wilcoxon rank-sum test) was conducted to identify potential differentially abundant features between the groups. Subsequently, linear discriminant analysis (LDA) was applied to the class labels on feature abundances to estimate the effect sizes for significant features. Only features with scaled LDA scores exceeding the threshold of 2.0 (default) were considered to be differentially abundant. This approach distinguishes LEfSe from standard Wilcoxon tests based on relative abundance. Notably, the raw LEfSe output does not undergo multiple-test correction, as only the *p*-values of significant features with above-threshold LDA scores are reported. For functional metagenomic analysis, the metagenome was reconstructed using phylogenetic investigation of communities by reconstruction of unobserved states (PICRUSt2) based on the operational taxonomic unit (OTU) table obtained from the 16S rRNA sequencing data [14]. All predicted functional genes were categorized into the Kyoto Encyclopedia of Genes and Genomes Orthology (KO) categories. Phylogenetic Investigation of Communities by Reconstruction of Unobserved States (PICRUSt2) in the QIIME2 plugin was performed to predict functional abundances and KEGG pathways. Statistical analysis of taxonomic and functional profiles (STAMP) was used for conducting statistical tests by false discovery rate (FDR) correlation and visualizing the profile of feature abundance generated by PICRUSt2.

## Decision tree analysis

### Statistical analysis

The 16S rDNA sequencing data were analysed using linear discriminant analysis effect size (LEfSe) to detect significant differences among the different groups. Spearman’s correlation analysis between gut microbiota and the expression of other genes was performed using Genescloud tools (https://www.genescloud.cn). All data are presented as mean ± standard error of the mean (SEM) and compared using one-way ANOVA and t-tests in SPSS 25.0 software (Chicago, USA) between experimental groups. Graphs were generated using Prism 9.0 software. Statistical significance was set at *p* < 0.05 was considered significant.

The performance of the predictive model was evaluated using a fivefold cross-validation approach and measured by the receiver operating characteristic (ROC) curve and area under the ROC curve (AUC). Critical gut microbiota data were further analysed using multivariate analyses in SPSS 25.0. False discovery rate (FDR) analysis was conducted using the Benjamini-Hochberg method, with adjusted p-values (q-values) less than 0.05 considered statistically significant. A Decision Tree (DT) algorithm was applied to identify critical predictors associated with Kawasaki disease. The non-parametric DT algorithm is composed of decision nodes (where data is split) and leaves (representing final outcomes or classifications), and the Chi-square Automatic Interaction Detector (CHAID) method was used to enhance the robustness and interpretability of the analysis. The hierarchy of prediction factors were presented and the most relevant variables for classification were indicated. The CHAID method was implemented using SPSS v25 to construct the tree, which split the data based on the most discriminative bacterial attributes.

## Results

### Characteristics of study participants

A total of 114 children were enrolled including 59 KD children (average age 1.4 ± 0.9 years; gender, male:female, 38:21) and 55 age matched control children (average age 2.5 ± 1.5 years; gender, male: female, 32:23) from Kaohsiung Chang Gung Memorial Hospital in Taiwan. There were no significant differences in BMI, body weight, or body height between children with KD and the controls (Supplementary Table S1).

### Differences of gut microbial alpha and beta diversity among acute KD children and controls

Raw sequences with a mean length of 550 bp were obtained from 114 faecal samples. After quality trimming and chimera checking 10,995,416 high-quality sequences remained, averaging 96,451 high-quality sequences per sample for downstream analysis. We identified 470 operational taxonomic units (OTUs) across all samples from the two groups: 269 OTUs in children with acute KD and 407 OTUs in the controls, following clustering at a 97% similarity level. These results suggest that the majority of faecal microbiota species were captured, indicating a robust sequencing depth for investigating faecal microbiota in children.

**Figure 1.**
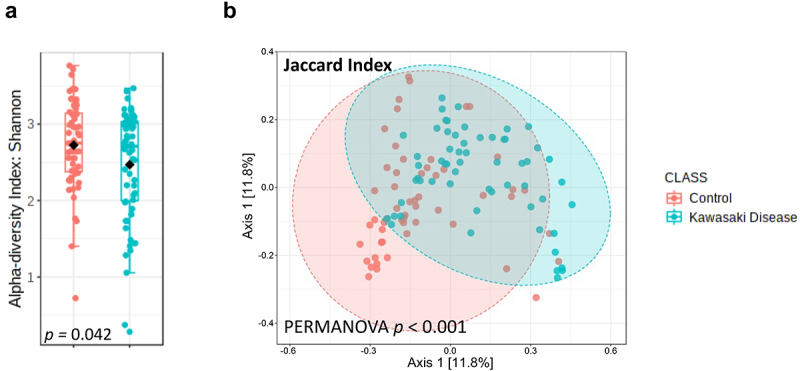
Gut microbiota composition change in Kawasaki disease (KD) patients. (a) Shannon alpha diversity (including richness and evenness) was significantly decreased in Kawasaki disease patients (*p* = 0.042). (b) Jaccard beta diversity bacteria composition was significantly changed in Kawasaki disease (*p* < 0.001). A *p* value less than 0.05 was considered statistically significant.

Alpha diversity indices were calculated to assess the overall faecal microbiota richness and evenness between the two groups. The Shannon diversity index showed a significant decrease in children with KD compared to that in controls (*p* = 0.042, Figure 1a). We also found that the Chao1 and ACE indices showed significantly reduced richness in KD patients compared to the control group (*p* < 0.001). However, the Simpson index did not show a significant difference, suggesting that while microbial richness is reduced, the evenness of the microbial community in KD patients may remain relatively unchanged (Supplementary Figure S1). Together, these findings indicate that KD is associated with a loss of microbial diversity and richness, which may contribute to the disease’s pathogenesis. The Jaccard index, also referred to as the Jaccard similarity coefficient, measures the similarity between two sets by comparing the size of their intersection to the size of their union. In Principal Coordinates Analysis (PCoA), the Jaccard index is utilized to evaluate the similarities or dissimilarities among different samples or objects. Therefore, to assess the similarity of the gut microbial communities between the two groups, Beta-diversity was evaluated using PCoA based on the Jaccard diversity index. This analysis revealed that the faecal microbiota of children with acute KD was distinct from that of controls (*p* < 0.001, PERMANOVA, Figure 1b). Additionally, beta-diversity was assessed using two different dissimilarity measures and statistical tests: PERMANOVA and ANOSIM (Supplementary Figure S2, top row and bottom row, respectively). The significant *p*-value <0.001 confirms that the differences between the KD and control groups exceed the variation within each group, supporting the robustness of the observed distinctions in microbial composition. These findings suggest that differences in gut microbiota composition and richness may play an important role in KD development.

### Differences in microbiota composition between acute KD and controls

**Figure 2.**
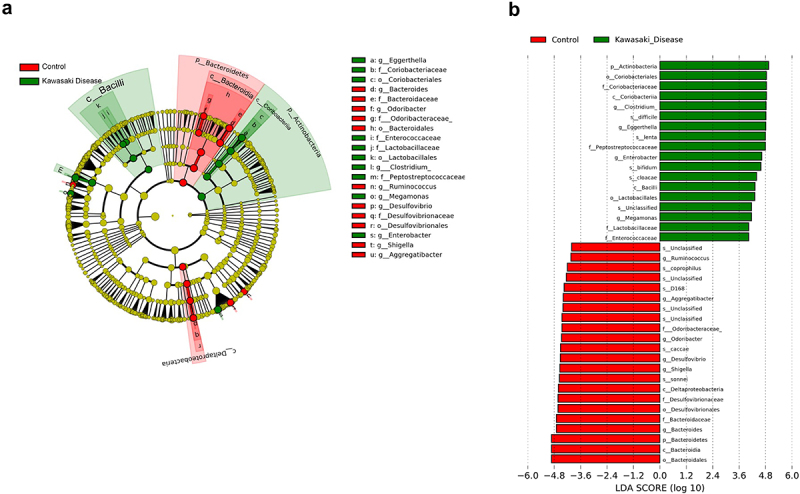
Core microbiota analysis between healthy control and Kawasaki disease. (a) the cladogram plots of core microbiota analysis. Red colour is presented healthy control. Green colour is presented Kawasaki disease patients. (b) LEfSe bar plots shown significantly changed gut microbiota in healthy control and Kawasaki disease. The cut-off value of LDA score is 4.0.

To identify potential bacterial biomarkers driving the differentiation of microbiota between children with KD and controls, we conducted LEfSe analysis at the genus level (LDA score > 4.0, *p* < 0.05; Figure 2a,b). After False Discovery Rate (FDR) correction using the Benjamini-Hochberg method, several genera were found to be significantly different between the two groups. Next, we identified the critical bacteria that might be involved in KD formation. Previous reports indicate that sex and age are significant factors influencing the composition of the gut microbiota [15,16]. After univariate and multivariate regression were used to identify the key factors involved in KD formation. Analysis of core microbiota identified significant differences between the Kawasaki disease group and the control group, particularly among species belonging to the *Firmicutes*, *Bacteroidetes*, and *Actinobacteria* phyla (Table 1 and Supplementary Table S2). In the *Firmicutes* phylum, several species were found to be elevated in the KD group. *Rothia mucilaginosa* (*p* < 0.001, FDR < 0.001, LDA score = 4.65) exhibited significantly higher abundance in KD patients compared to controls. *Clostridium difficile* (*p* < 0.001, FDR < 0.001, LDA score = 4.59), *Eggerthella lenta* (*p* < 0.001, FDR < 0.001, LDA score = 4.31) and *Collinsella aerofaciens* (*p* = 0.013, FDR = 0.020, LDA score = 4.33) were also enriched in KD patients. Conversely, *Faecalibacterium prausnitzii* (*p* = 0.002, FDR = 0.004, LDA score = −4.56) showed reduced abundance in the KD group compared to controls. Within the *Actinobacteria* phylum, *Bifidobacterium* species were notably more abundant in the KD group. *Bifidobacterium longum* (*p* < 0.001, FDR < 0.001, LDA score = 5.55) exhibited the highest enrichment in KD patients. Other species in the same genus, such as *Bifidobacterium bifidum* (*p* < 0.001, FDR < 0.001, LDA score = 4.36) and *Bifidobacterium adolescentis* (*p* = 0.004, FDR = 0.008, LDA score = 4.20), were also significantly increased in KD patients.In contrast, species from the *Bacteroidetes* phylum showed a consistent reduction in the KD group. *Bacteroides ovatus* (*p* < 0.001, FDR < 0.001, LDA score = −5.43) and *Bacteroides uniformis* (*p* < 0.001, FDR < 0.001, LDA score = −4.92) were significantly depleted in KD patients. Similarly, *Bacteroides fragilis* (*p* = 0.009, FDR = 0.015, LDA score = −5.26) and *Bacteroides caccae* (*p* = 0.005, FDR = 0.008, LDA score = −4.41) demonstrated lower abundance in the KD group. Prevotella copri (*p* = 0.022, FDR = 0.032, LDA score = −4.84) was also significantly reduced. Furthermore, several bacterial species exhibited strong associations with KD in the univariate analysis (Table 2, upper panel). Notably, *Eggerthella lenta* showed a highly significant positive association with Kawasaki disease (*p* < 0.001), with an odds ratio (OR) of 2.984 × 10^5^ and a 95% confidence interval (CI) ranging from 2.900 × 10^2^ to 3.070 × 10^8^. This indicates that the presence of *Eggerthella lenta* is markedly elevated in KD patients compared to controls. Similarly, *Clostridium difficile* was significantly enriched in KD patients (*p* = 0.006, OR = 18.931, 95% CI = 2.361 to 151.756). Conversely, *Bacteroides ovatus* showed a significant negative association with KD (*p* = 0.004, OR = 0.849, 95% CI = 0.760 to 0.949), indicating that this species is significantly depleted in KD patients. Additionally, *Faecalibacterium prausnitzii* was reduced in KD patients (*p* = 0.016, OR = 0.651, 95% CI = 0.460 to 0.922), reflecting a potential loss of beneficial microbial activity. *Bifidobacterium longum* was positively associated with KD (*p* = 0.002, OR = 1.273, 95% CI = 1.096 to 1.478), suggesting an increased abundance in KD patients. In the multivariate analysis, after adjusting for potential confounding factors, *Eggerthella lenta* maintained a strong and significant positive association with Kawasaki disease (*p* = 0.014, OR = 1.702 × 10^4^, 95% CI = 6.997 to 4.142 × 10^7^) (Table 2, lower panel). This reinforces the potential role of *Eggerthella lenta* in the pathogenesis of KD. *Bacteroides ovatus* also retained its significant negative association (*p* = 0.016, OR = 0.778, 95% CI = 0.635 to 0.954), indicating that its depletion may be consistently linked to KD. In contrast, the association of *Clostridium difficile* was no longer significant in the multivariate analysis (*p* = 0.125), suggesting that its relationship with KD may be influenced by other variables. Overall, these findings indicate a distinctive pattern of dysbiosis in Kawasaki disease, characterized by the enrichment of *Eggerthella lenta* and the depletion of *Bacteroides ovatus*. The consistent association of *Eggerthella lenta* with KD suggests its potential involvement in inflammatory processes, while the reduction of *Bacteroides ovatus* may reflect a loss of microbial species that play a role in maintaining gut homoeostasis and immune regulation.

**Table 1.** Core microbiota in species level with FDR (LDA score ≥4.0).

	Control	Kawasaki Disease		
	Mean ± SEM	Mean ± SEM	*p* value	*q value*
**Phylum**				
*Firmicutes*	15590 ± 40010.39	41017.86 ± 3340.89	*0.003*	*0.004*
*Bacteroidetes*	36905.87 ± 9785.84	29037.56 ± 3602.10	*<0.001*	*<0.001*
*Actinobacteria*	1649.85 ± 586.97	15435.27 ± 1992.71	*<0.001*	*<0.001*
**Family**				
*Enterococcaceae*	87.13 ± 36.05	5449.22 ± 2181.58	*<0.001*	*<0.001*
*Actinomycetaceae*	3.40 ± 1.73	154.25 ± 79.78	*<0.001*	*<0.001*
*Micrococcaceae*	10.22 ± 6.28	824.85 ± 558.77	*<0.001*	*<0.001*
*Coriobacteriaceae*	76.02 ± 36.28	1257.29 ± 368.51	*<0.001*	*<0.001*
*Bacteroidaceae*	34912.56 ± 9664.63	24581.12 ± 3300.71	*<0.001*	*<0.001*
*Bifidobacteriaceae*	1559.93 ± 566.57	13185.20 ± 1883.70	*<0.001*	*<0.001*
*Peptostreptococcaceae*	99.38 ± 54.80	931.34 ± 252.91	*<0.001*	*<0.001*
*Enterobacteriaceae*	5002.56 ± 1473.80	29927.69 ± 3790.60	*<0.001*	*<0.001*
*Carnobacteriaceae*	30.49 ± 14.24	214.20 ± 122.26	*<0.001*	*<0.001*
**Genus**				
*Salmonella*	0.75 ± 0.43	17.07 ± 4.98	*<0.001*	*<0.001*
*Enterococcus*	85.58 ± 35.51	5405.42 ± 2179.70	*<0.001*	*<0.001*
*Morganella*	10.96 ± 6.96	108.05 ± 41.73	*<0.001*	*<0.001*
*Eggerthella*	35.69 ± 21.47	488.86 ± 113.58	*<0.001*	*<0.001*
*Actinomyces*	3.40 ± 1.73	151.27 ± 79.78	*<0.001*	*<0.001*
*Rothia*	2.47 ± 1.59	822.88 ± 558.33	*<0.001*	*<0.001*
*Bacteroides*	34912.56 ± 9664.63	24581.10 ± 3300.71	*<0.001*	*<0.001*
*Bifidobacterium*	1559.91 ± 566.57	13185.12 ± 1883.70	*<0.001*	*<0.001*
*Clostridium*	123.91 ± 58.46	130.41 ± 47.36	*<0.001*	*<0.001*
**Species**				
*Rothia mucilaginosa*	0.003 ± 0.001	0.720 ± 0.506	*0.174*	*0.244*
*Faecalibacterium prausnitzii*	1.399 ± 0.341	0.389 ± 0.134	*0.006*	*0.013*
*Eubacterium dolichum*	0.099 ± 0.028	1.594 ± 0.418	*<0.001*	*0.004*
*Eggerthella lenta*	0.018 ± 0.008	0.365 ± 0.090	*<0.001*	*0.002*
*Clostridium difficile*	0.047 ± 0.019	0.738 ± 0.207	*0.002*	*0.005*
*Bifidobacterium longum*	1.048 ± 0.375	7.629 ± 1.363	*<0.001*	*<0.001*
*Prevotella copri*	1.711 ± 1.283	0.327 ± 0.183	*0.271*	*0.304*
*Bacteroides uniformis*	3.288 ± 0.869	1.759 ± 0.517	*0.127*	*0.205*
*Bifidobacterium adolescentis*	0.243 ± 0.177	0.528 ± 0.264	*0.378*	*0.378*
*Bacteroides ovatus*	5.450 ± 1.120	1.404 ± 0.540	*0.001*	*0.004*
*Collinsella aerofaciens*	0.095 ± 0.048	0.477 ± 0.238	*0.132*	*0.205*
*Bifidobacterium bifidum*	0.079 ± 0.046	0.480 ± 0.157	*0.019*	*0.038*
*Bacteroides fragilis*	9.037 ± 2.370	5.777 ± 1.463	*0.237*	*0.302*
*Bacteroides caccae*	1.075 ± 0.334	0.634 ± 0.242	*0.282*	*0.304*

**Table 2.** Univariant and multivariant regression.

	*p value*	Odds Ratio	95% CI
**Univariant**			
Age	*0.011*	0.711	0.547 to 0.925
Gender (Male = 1, Female = 0)	*0.495*	0.796	0.361 to 1.637
*Faecalibacterium prausnitzii*	*0.016*	0.651	0.460 to 0.922
*Eubacterium dolichum*	*0.004*	11.067	2.149 to 56.996
*Eggerthella lenta*	*<0.001*	2.984 * 10^5^	2.900 * 10^2^ to 3.070 * 10^8^
*Clostridium difficile*	*0.006*	18.931	2.361 to 151.756
*Bifidobacterium longum*	*0.002*	1.273	1.096 to 1.478
*Bacteroides ovatus*	*0.004*	0.849	0.760 to 0.949
*Bifidobacterium bifidum*	*0.055*	2.974	0.976 to 9.061
**Multivariant**			
Age	*0.700*	0.934	0.661 to 1.321
*Faecalibacterium prausnitzii*	*0.348*	0.821	0.544 to 1.239
*Eubacterium dolichum*	*0.055*	4.463	0.968 to 20.590
*Eggerthella lenta*	*0.014*	1.702 * 10^4^	6.997 to 4.142 * 107
*Clostridium difficile*	*0.125*	4.239	0.669 to 26.847
*Bifidobacterium longum*	*0.121*	1.080	0.980 to 1.192
*Bacteroides ovatus*	*0.016*	0.778	0.635 to 0.954

**Figure 3.**
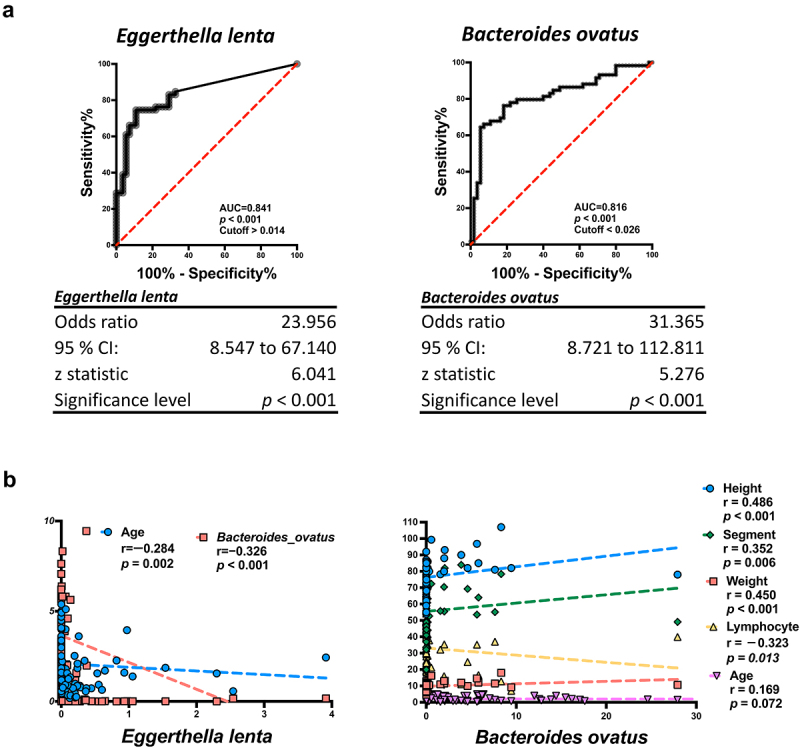
*Eggerthella lenta* and *Bacteroides ovatus* are significantly changed in Kawasaki disease. (a) receiver operating characteristic (ROC) curve were used to analyse the *Eggerthella lenta* and *Bacteroides ovatus* and get the cut-off value (>0.041 and < 0.026, respectively) and area under curve (0.841 and 0.816, respectively). (b) the correlation plot between *Eggerthella lenta* and B*acteroides ovatus*. *Eggerthella lenta* shown negative correlation with age and *Bacteroides ovatus* (*r* = −0.284, *p* = 0.002, and *r* = −0.326, *p* < 0.001, respectively). *Bacteroides ovatus* shown positive correlation with blood segments cells (*r* = 0.352, *p* = 0.006) and negative correlation with blood lymphocytes (*r* = −0.323, *p* = 0.013). A *p* value less than 0.05 was considered statistically significant.

The area under the receiver operating characteristic curve (AUC) of *Eggerthella lenta* and *Bacteroides ovatus* was 0.841 and 0.816, respectively (Figure 3a). The cut-off values for these two bacteria were 0.014 and 0.026, respectively. These results indicated that *Eggerthella lenta* and *Bacteroides ovatus* are biomarkers of KD. To understand the role of these two bacteria in Kawasaki Disease, we performed Spearman’s correlation analysis between these bacteria and various biochemical and physiological parameters. *Eggerthella lenta* was negatively correlated with age and *Bacteroides ovatus* (*r* = −0.284, *p* = 0.002 and *r* = −0.326, *p* < 0.001, respectively; Figure 3b). Interestingly, the well-known probiotics *Bifidobacterium longum* and *Bifidobacterium bifidum*, were increased in the KD group (*p* < 0.001, q < 0.001 and *p* < 0.001, q = 0.038, respectively; Supplementary Figure S3A). These results might be due to the stress response effects in KD [17]. *Eggerthella lenta* was negatively correlated with *Bacteroides ovatus*, and the ratio of *Eggerthella lenta* to *Bacteroides ovatus* showed a significant increase in KD (*p* < 0.001; Supplementary Figure S3B, upper panel). The AUC of this ratio was 0.859 (*p* < 0.001) and the cut-off value was 0.018 (Supplementary Figure S3C). The ratio of age-adjusted *Eggerthella lenta* was significantly increased in KD (*p* < 0.001, Supplementary Figure S3D), and the AUC was 0.845 (*p* < 0.001, Supplementary Figure S3E). This result indicated that *Eggerthella lenta* could be a predictive marker of KD. Furthermore, *Bacteroides ovatus* showed a positive correlation with body height, body weight, and blood segments (*r* = 0.486, *p* < 0.001; *r* = 0.450, *p* < 0.001; and *r* = 0.352, *p* = 0.006, respectively) and a negative correlation with blood lymphocytes (*r* = −0.323, *p* = 0.013; Figure 3b, lower panel). These two bacteria may influence the behaviour of blood immune cells and contribute to the development of Kawasaki Disease.

### Eggerthella lenta and Bacteroides ovatus influence flavone and flavonol biosynthesis signalling pathway in Kawasaki Disease

**Figure 4.**
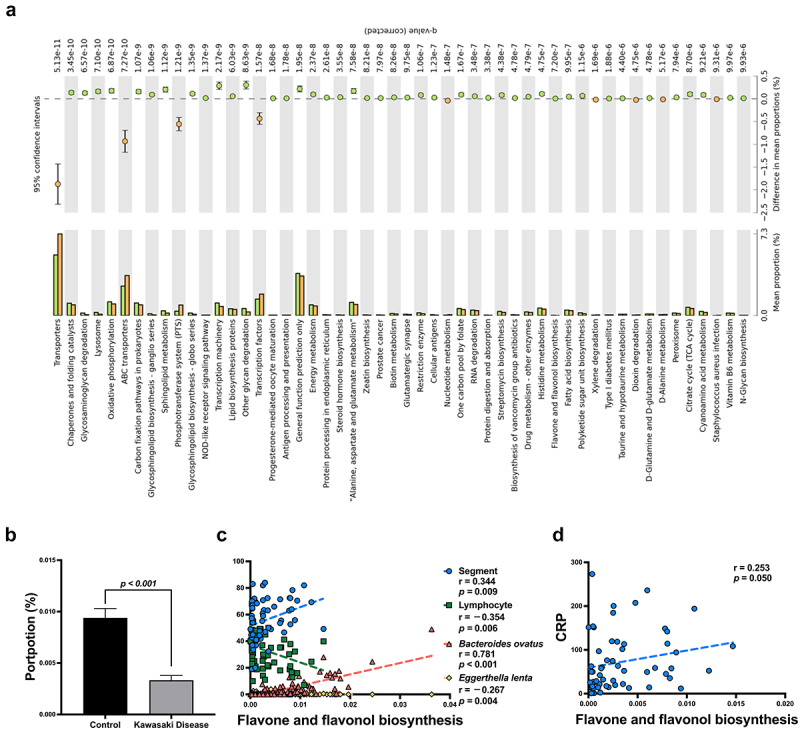
The critical signalling pathway in Kawasaki disease is flavone and flavonol biosynthesis. a. functional pathways analysis by PICRUSt and false positive discovery rate (FDR) analysis was utilized by Benjamini-Hochberg method and adj *p*-value (*q* value) less than 0.05 were consider as statistically significant. b. flavone and flavonol biosynthesis was downregulated in Kawasaki disease. c. flavone and flavonol biosynthesis shown negative correlation with *Eggerthella lenta* and blood lymphocytes (r = −0.267, *p* = 0.004 and *r* = −0.354, *p* = 0.006, respectively); and shown positive correlation with *bacteroides ovatus* and blood segments cells (r = 0.781, *p* < 0.001, and r = 0.344, *p* = 0.009, respectively). d. flavone and flavonol biosynthesis shown positive correlation with C-reactive protein (CRP, r = 0.253, *p* = 0.050). A *p* value less than 0.05 was considered statistically significant.

To further investigate which signalling pathways might be involved in KD formation, Phylogenetic Investigation of Communities by Reconstruction of Unobserved States 2 (PICRUSt2) was used with FDR correlation to analyse potential functional pathways (see Figure 4a). Significant functional pathways were further analysed using a univariate regression model in different KEGG Level 2 categories. In total, ten functional pathways exhibited significant changes in KD (Table 3). However, the flavone and flavonol biosynthesis pathway was the only signalling pathway that was significantly decreased in KD, according to multivariate regression (*p* < 0.001; see Figure 4b and Table 3). This pathway showed a negative correlation with *Eggerthella lenta* and blood lymphocytes (*r* = −0.267, *p* = 0.004 and *r* = −0.354, *p* = 0.006, respectively), and a positive correlation with *Bacteroides ovatus* and blood segments (*r* = 0.781, *p* < 0.001 and *r* = 0.334, *p* = 0.009, respectively; Figure 4c). Blood lymphocytes were negatively correlated with CRP (*r* = −0.443, *p* < 0.001), and blood segments showed a positive correlation with CRP (*r* = 0.496, *p* < 0.001, Supplementary Figure S4A). Moreover, the ratio of *Eggerthella lenta* to *Bacteroides ovatus* was negatively correlated with flavone and flavonol biosynthesis (*r* = −0.295, *p* = 0.025 and *r* = −0.465, *p* < 0.001, respectively; Supplementary Figure S4B). This result suggests that *Eggerthella lenta* and *Bacteroides ovatus* might influence the regulation of blood segments and lymphocytes through the biosynthesis of flavones and flavonols, potentially playing a role in the incidence of Kawasaki Disease. The clinical laboratory data of KD patients were shown in Supplementary Table S3. However, no significant association was observed between CRP levels and the biosynthesis of flavones and flavonols (Figure 4d). We further employed the Decision Tree (DT) algorithm with the Chi-square Automatic Interaction Detector (CHAID) method to identify critical bacterial predictors associated with KD. The decision tree was constructed using gut bacterial data identified through univariate and multivariate regression analyses, and the *Eggerthella lenta* to *Bacteroides ovatus* ratio was used as the potential predictor.

**Table 3.** Multivariant regression.

Pathways	*p value*	Odds Ratio	95 % CI
Flavone and flavonol biosynthesis	*0.003*	0	0
NOD-like receptor signalling pathway	*0.921*	93.833	0 to 1.130 * 10^41^
Energy metabolism	*0.811*	0.238	0 to 31,756.775
Biotin metabolism	*0.971*	0.428	0 to 1.421 * 10^19^
Isoquinoline alkaloid biosynthesis	*0.445*	9.117 * 10^16^	0 to 2.780 * 10^60^
C5-Branched dibasic acid metabolism	*0.151*	2.660 * 10^8^	0.001 to 8.149 * 10^19^
Glycolysis/Gluconeogenesis	*0.112*	1.665 * 10^4^	0.105 to 2.635 * 10^9^
Oxidative phosphorylation	*0.114*	0	0 to 6.686
Selenocompound metabolism	*0.800*	0.002	0 to 5.202 × 10^17^
Steroid hormone biosynthesis	*0.766*	0.015	0 to 1.650 × 10^10^

**Figure 5.**
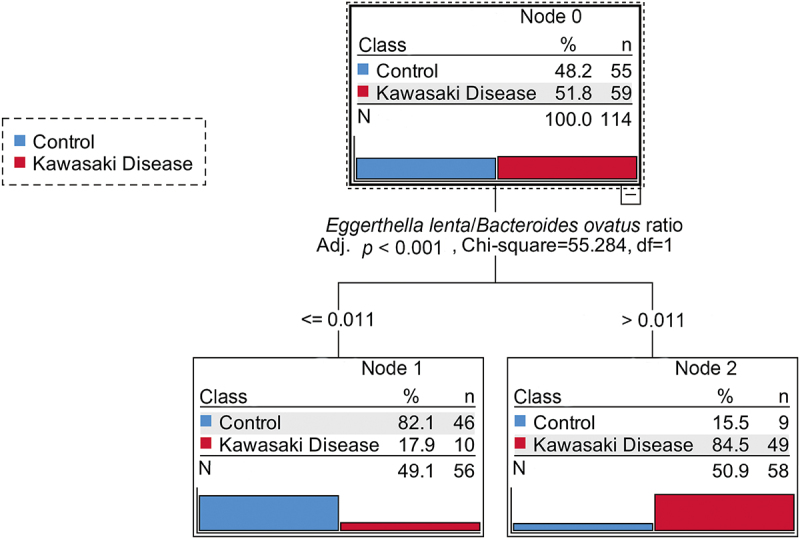
Decision tree analysis for Kawasaki disease prediction based on the *Eggerthella lenta*/*Bacteroides ovatus* ratio. the root node shows 114 samples (55 controls and 59 KD patients), with a split at a threshold ratio of 0.011 (adjusted *p* < 0.001, chi-square = 55.284, df = 1). For samples with a ratio of 0.011 or lower, 82.1% are classified as controls (node 1), while for samples with a ratio greater than 0.011, 84.5% are classified as KD patients (node 2). A *p* value less than 0.05 was considered statistically significant.

As shown in Figure 5, the decision tree analysis stratified the samples based on the *Eggerthella lenta*/*Bacteroides ovatus* ratio. For samples with a ratio of 0.011 or lower (Node 1), 82.1% were classified as controls (46 out of 56), while 17.9% were classified as KD patients (10 out of 56). In contrast, for samples with a ratio greater than 0.011 (Node 2), 84.5% were classified as KD patients (49 out of 58), while 15.5% were controls (9 out of 58). The split at the threshold of 0.011 effectively separated the samples into two distinct groups, which was statistically significant for KD prediction (adjusted *p* < 0.001, Chi-square = 55.284). These results demonstrated that an elevated *Eggerthella lenta*/*Bacteroides ovatus* ratio is a robust predictor of KD. The threshold indicates the potential utility of the *Eggerthella lenta*/*Bacteroides ovatus* ratio as a biomarker for KD. The findings suggest that an overabundance of *Eggerthella lenta* relative to *Bacteroides ovatus* may play a role in the pathogenesis of KD.

## Discussion

KD is a paediatric, self-limiting, systemic inflammatory vasculitis that was first described in 1967 in Japan by Dr. Tomisaku Kawasaki [18]. Gut microbiota has been emphasized in immune response regulation [19,20]. In our findings, alpha diversity was observed to decrease in KD. Alpha diversity serves as a key indicator for assessing the diversity of the gut microbiota. Notably, lower alpha diversity in the gut microbiota has been linked to various diseases such as obesity, diabetes, and colorectal cancer [21–23]. The composition of the gut microbiota (beta diversity) was significantly different between KD patients and healthy controls. These findings suggested that gut dysbiosis is associated with KD.

Core bacteria were analysed by the LEfSe method and seven bacteria were (5 beneficial bacteria, such as *Faecalibacterium prausnitzii, Eubacterium dolichum, Bifidobacterium longum, Bacteroides ovatus*, and B*ifidobacterium bifidum* and 2 harmful bacteria, such as *Eggerthella lenta* and *Clostridium difficile*) were significantly changed in KD. *Clostridium difficile*, the most important gastrointestinal disease, was significantly increased in KD patients. *Clostridium difficile* has been reported in children and infants with KD [24]. The mechanism of action of these bacteria in KD requires further investigation. *Eggerthella lenta* significantly increased in patients with KD. This bacterium has been implicated in IgA nephropathy (IgAN), caused by dysbiosis [25,26].

Our results provide evidence for the role of the prevalent human gut, *Eggerthella lenta and Bacteroides ovatus*, in KD. *Eggerthella lenta* was significantly enriched in KD patients and may contribute to inflammation through multiple pathways. This non-spore-forming Gram-positive, anaerobic bacillus is known to be among the natural members of the gut microbiota, and has been focused on its role in bacteraemia [27]. Recent studies have shown that the abundance of *Eggerthella* is significantly elevated in various immune-mediated inflammatory diseases, including Crohn’s disease, ulcerative colitis, multiple sclerosis, rheumatoid arthritis, and Behçet’s disease, compared to healthy controls [28,29]. *Eggerthella lenta* has also been associated with multiple sclerosis and rheumatoid arthritis [9,30]. A notable increase in *Eggerthella*, particularly at the genus level and the species level (*Eggerthella lenta*), has also been observed in children with multisystem inflammatory syndrome (MIS-C) [31]. Additionally, *Eggerthella lenta* is involved in the metabolism of dietary compounds and bile acids, producing metabolites that may exacerbate Th17 activation [32]. Given the role of autoimmunity and the Th17 pathway in the pathogenesis of both KD and MIS-C, our findings indicate that an increased abundance of *Eggerthella lenta* may play a significant role in the pathogenesis of these diseases. The positive correlation between *Eggerthella lenta* abundance and elevated C-reactive protein (CRP) levels suggests its role in promoting systemic inflammation. The enrichment of this species in KD patients may thus reflect a dysbiotic state that favours pro-inflammatory pathways and contributes to the acute-phase response characteristic of KD.

In contrast, *Bacteroides ovatus* was significantly depleted in KD patients, and its reduced abundance may impair anti-inflammatory mechanisms. *Bacteroides ovatus*, a Gram-negative anaerobe that produces short-chain fatty acids (SCFAs) such as butyrate and propionate [33]. These SCFAs are critical for maintaining immune homoeostasis by promoting the differentiation of regulatory T cells (Tregs) and suppressing the production of pro-inflammatory cytokines [34]. The loss of *Bacteroides ovatus* may therefore reduce SCFA availability, leading to uncontrolled inflammation. This helps in maintaining a balanced immune response and preventing excessive inflammation. Additionally, *Bacteroides ovatus* plays a role in the metabolism of flavonoids [35]. The gut microbiota, including *Bacteroides ovatus*, plays a crucial role in converting dietary flavonoids into various metabolites, enhancing their health-promoting effect. Decreased levels of this species could impair flavone and flavonol biosynthesis, diminishing their protective effects against oxidative stress and inflammation. Furthermore, *Bacteroides ovatus* supports gut barrier integrity by enhancing mucin production and preserving tight junction proteins. When *Bacteroides ovatus* is depleted, it may lead to increase gut permeability, allowing harmful substances like lipopolysaccharides (LPS) to enter the bloodstream and trigger systemic inflammation [36]. *Bacteroides ovatus*, had been considered as a next-generation probiotic that could prevent LPS-induced inflammation and intestinal microbiota disorder in mice [37]. *Bacteroides ovatus* also plays a role in xylan metabolism, thereby constraining the development of colitis [38]. According to the multivariate regression analysis model, *Eggerthella lenta* and *Bacteroides ovatus* were identified as the primary bacterial species associated with Kawasaki disease (KD) development. The receiver operating characteristic (ROC) curve analysis further demonstrated that both *Eggerthella lenta* and *Bacteroides ovatus* are potential risk factors for KD. While the ratio of *Eggerthella lenta* to *Bacteroides ovatus* was significantly elevated in KD patients, it did not show improved predictive power (Supplementary Figure S3). *Eggerthella lenta* has previously been implicated in autoimmune diseases [32,39], suggesting its particular relevance in KD pathogenesis. Notably, *Eggerthella lenta* levels were negatively correlated with both *Bacteroides ovatus* abundance and age. In contrast, *Bacteroides ovatus* showed a negative correlation with blood lymphocyte levels [30,40]. These findings suggest that increased *Eggerthella lenta* and decreased *Bacteroides ovatus* may contribute to elevated blood lymphocyte counts during the immune response. This observation aligns with prior studies indicating that blood lymphocyte levels are elevated in KD patients [41,42]. The opposing roles of these two species indicate the dysbiotic state present in KD patients, where an imbalance between pro-inflammatory bacteria (*Eggerthella lenta*) and anti-inflammatory bacteria (*Bacteroides ovatus*) may drive systemic inflammation. The negative correlation between the *Eggerthella lenta/Bacteroides ovatus* ratio and CRP levels further supports this hypothesis (Supplementary Figure S4B). This imbalance may exacerbate the immune dysregulation and tissue damage characteristic of KD.

Flavones and flavonols are two subclasses of flavonoids that are plant-derived compounds known for their diverse biological activities, including potential effects on the immune system. The flavone and flavonol biosynthesis pathway showed a dramatic decrease in KD and was positively correlated with *Bacteroides ovatus* and blood segments, but negatively correlated with *Eggerthella lenta* and blood lymphocytes. The *Eggerthella lenta*-to-*Bacteroides ovtaus* ratio also showed a negative correlation with flavone and flavonol biosynthesis (Supplementary Figure S4C). In previous studies, flavones were shown to regulate the activity of intestinal immunity in patients with chronic bowel disease [43]. This compound acts through the mTOR signalling pathway in CD4+ regulatory T cells to regulate immunity. Our results indicate that the gut microbiota could also regulate flavone and flavonol biosynthesis, thereby influencing the immune system and potentially promoting KD development [44]. Flavones and flavonols are both potent antioxidants. They can scavenge free radicals and reduce oxidative stress, which is implicated in the development and progression of cardiovascular diseases, such as atherosclerosis. These compounds may help to maintain cardiovascular health by protecting cells from oxidative damage. Flavones and flavonols may exert cardioprotective effects through multiple mechanisms, including antioxidant, anti-inflammatory, vasodilatory, antiplatelet, and lipid-lowering effects. Regular intake of these compounds, which are typically found in fruits, vegetables, tea, and red wine, has been associated with a lower incidence of cardiovascular events in epidemiological studies. Quercetin (, a flavonoid) inhibits both NLRP3 and AIM2 inflammasomes by preventing apoptosis-associated speck-like protein containing a caspase recruitment domain (CARD) (ASC) oligomerization, and may be a potential therapeutic candidate for KD vasculitis and other IL-1 mediated inflammatory diseases [45]. Portman also reported that isoflavones participate in KD pathogenesis by modulating the function of FcGRs and disrupting the balance between activation and inhibition of the inflammatory response [46]. However, the lack of correlation between CRP levels and the flavone and flavonol biosynthesis pathway indicating that this pathway is not the sole driver of systemic inflammation in Kawasaki disease. Systemic inflammation in KD is complex and likely driven by multiple pathways and factors [47]. While the flavone and flavonol biosynthesis pathway may play a role in modulating inflammation through antioxidant and anti-inflammatory effects [48], it is unlikely to be the sole contributor. Other pathways, such as those involved in the production of cytokines, acute-phase proteins, or other inflammatory mediators, may also play dominant roles in elevating CRP levels. The flavone and flavonol biosynthesis pathway could exert indirect or localized effects on inflammation that are not immediately reflected in systemic CRP levels. This pathway is known for producing compounds with antioxidant and anti-inflammatory properties, which may act on local tissues (e.g. gut or vascular endothelium) rather than systemically. Instead, CRP levels may be more responsive to other inflammatory triggers, such as microbial dysbiosis, immune cell activation, or vascular damage associated with KD. Moreover, *Bifidobacterium longum* and *Bifidobacterium bifidum* have been considered probiotics in several diseases, such as obesity [49], inflammatory bowel disease [50], atopic dermatitis [51], and cardiovascular diseases [52]. In this study, *Bifidobacterium longum* and *Bifidobacterium bifidum* were significantly increased in KD. Recent studies have mentioned that beneficial bacteria, such as *Bifidobacterium spp*., can modulate inflammatory responses through downregulated interferon-gamma production in mice [53]. These results indicate that early *Bifidobacterium spp*. induction may play a protective role in KD but still requires further investigation. In the univariate regression model, age was one of the risk factors for disease formation in KD. This result is consistent with previous studies in children under 5 years of age as a risk factor for KD [54].

In summary, gut dysbiosis in KD emerges as a significant risk factor in patients with KD. Specifically, an increase in *Eggerthella lenta* and a decrease in *Bacteroides ovatus* are associated with downregulation of the flavone and flavonol biosynthesis pathways. This dysregulation in turn promotes immune responses. This study marks the first report linking the flavone and flavonol biosynthesis pathways with the microbiota in the immunopathogenesis of KD. This suggests that changes in the gut microbiota offer a new perspective for understanding the causes and mechanisms of KD. The small sample size and cross-sectional design limit the statistical power and generalizability of our findings, restricting conclusions on causality and the influence of confounding factors like diet and antibiotic use. Future research should involve larger, multi-centre, and longitudinal studies, integrating multi-omics approaches and functional validation to better understand the interplay between microbial composition, metabolic pathways, and host immunity. Despite these limitations, our current work highlights significant microbial and metabolic differences in Kawasaki disease, offering valuable insights into potential biomarkers and pathways that may inform future diagnostics and therapeutic strategies.

## Supplementary Material

Supplementary Tables.docx

Supplementary Figure 3.tif

Supplementary Figure 4.tif

Supplementary Figures and Legends.docx

Supplementary Figure 2.tif

Supplementary Figure 1.tif

## Data Availability

All data generated or analysed during this study are publicly available in recognized repositories. The raw sequencing data are accessible in the SRA Run Selector under project ID PRJNA1207020 (https://www.ncbi.nlm.nih.gov/Traces/study/?acc=PRJNA1207020). Processed data, including those used to generate all figures, tables, and statistical analyses in the manuscript, are available in the GEO repository under accession number GSE285935 (https://www.ncbi.nlm.nih.gov/geo/query/acc.cgi?acc=GSE285935).

## Abbreviations

KD: Kawasaki disease,
IVIG: intravenous immunoglobulin,
CAL: coronary artery lesions, complete blood count with differential count (CBC/DC),
CRP: C-reactive protein
ALT: alanine aminotransferase
