# Supplementary material for: Eggerthella lenta down regulated flavone and flavonol biosynthesis promoted Kawasaki disease
Source: Virulence. 2025 May 31;16(1):2512401. doi: 10.1080/21505594.2025.2512401 (PMC12128670; doi:10.1080/21505594.2025.2512401)
Supplement: Supplementary Tables.docx [file KVIR_A_2512401_SM6176.docx]

**Supplementary Table 1.** Core microbiota Genus level with FDR (LDA Score ≥ 4.0)

| **Genus level** | ***p* values** | **FDR (*q* value)** | **LDA score** |
| --- | --- | --- | --- |
| *Bifidobacterium spp.* | <0.001 | <0.001 | 5.66 |
| *Enterococcus spp.* | <0.001 | <0.001 | 5.32 |
| *Klebsiella spp.* | <0.001 | <0.001 | 4.94 |
| *Eubacterium spp.* | <0.001 | <0.001 | 4.93 |
| *Streptococcus spp.* | <0.001 | <0.001 | 4.81 |
| *Rothia spp.* | <0.001 | <0.001 | 4.65 |
| *Clostridium spp.* | <0.001 | <0.001 | 4.59 |
| *Blautia spp.* | 0.008 | 0.013 | 4.58 |
| *Collinsella spp.* | 0.013 | 0.020 | 4.33 |
| *Eggerthella spp.* | <0.001 | <0.001 | 4.31 |
| *Faecalibacterium spp.* | 0.002 | 0.004 | -4.56 |
| *Oscillospira spp.* | 0.005 | 0.009 | -4.56 |
| *Prevotella spp.* | 0.022 | 0.031 | -4.84 |
| *Akkermansia spp.* | 0.441 | 0.478 | -4.91 |
| *Bacteroides spp.* | <0.001 | <0.001 | -6.22 |

**Supplementary Table 2.** Core microbiota in Species level with FDR (LDA Score ≥ 4.0)

| **Species** | ***p* values** | **FDR** | **LDA Score** |
| --- | --- | --- | --- |
| *Eggerthella lenta* | <0.001 | <0.001 | 4.31 |
| *Rothia mucilaginosa* | <0.001 | <0.001 | 4.65 |
| *Bacteroides ovatus* | <0.001 | <0.001 | -5.43 |
| *Bifidobacterium longum* | <0.001 | <0.001 | 5.55 |
| *Clostridium difficile* | <0.001 | <0.001 | 4.59 |
| *Eubacterium dolichum* | <0.001 | <0.001 | 4.93 |
| *Bifidobacterium bifidum* | <0.001 | <0.001 | 4.36 |
| *Bacteroides uniformis* | <0.001 | <0.001 | -4.92 |
| *Faecalibacterium prausnitzii* | 0.002 | 0.004 | -4.56 |
| *Bifidobacterium adolescentis* | 0.004 | 0.008 | 4.20 |
| *Bacteroides caccae* | 0.005 | 0.008 | -4.41 |
| *Bacteroides fragilis* | 0.009 | 0.015 | -5.26 |
| *Collinsella aerofaciens* | 0.013 | 0.020 | 4.33 |
| *Prevotella copri* | 0.022 | 0.032 | -4.84 |

**Supplementary Table 3.** Clinical laboratory data of Kawasaki disease

| **Items** | **Mean ± SEM** |
| --- | --- |
| ESR (mm/hr) | 50.15 ± 4.03 |
| WBC (1000/ml) | 13.17 ± 0.50 |
| RBC (10⁶/ml) | 4.36 ± 0.07 |
| Hemoglobin (g/dl) | 11.04 ± 0.13 |
| Hematocrit (%) | 33.72 ± 0.37 |
| MCV (fl) | 77.87 ± 0.86 |
| MCH (pg/cell) | 25.51 ± 0.33 |
| MCHC (gHb/dL) | 32.72 ± 0.15 |
| RDW-SD (fl) | 37.83 ± 0.40 |
| Platelets (10³/ml) | 337.47 ± 15.01 |
| RDW-CV (%) | 13.48 ± 0.15 |
| Segment (%) | 56.25 ± 1.96 |
| Lymphocyte (%) | 32.57 ± 1.69 |
| Monocyte (%) | 5.97 ± 0.35 |
| Eosinophil (%) | 3.85 ± 0.42 |
| Basophil (%) | 0.19 ± 0.03 |
| AST (U/L) | 82.42 ± 19.76 |
| ALT (U/L) | 88.21 ± 18.39 |
| CRP (mg/L) | 72.35 ± 8.50 |
| Sodium (mEq/L) | 136.20 ± 0.36 |
| Alb. (g/dL) | 3.91 ± 0.06 |
