# Supplementary material for: Eggerthella lenta down regulated flavone and flavonol biosynthesis promoted Kawasaki disease
Source: Virulence. 2025 May 31;16(1):2512401. doi: 10.1080/21505594.2025.2512401 (PMC12128670; doi:10.1080/21505594.2025.2512401)
Supplement: Supplementary Figures and Legends.docx [file KVIR_A_2512401_SM6173.docx]

**Supplementary Figures and Legends**

**Supplementary Figure 1. Comparison of Alpha Diversity Indices Between Kawasaki Disease and Control Groups.** Alpha diversity was analyzed using four additional indices: Chao1, ACE, Simpson, and Fisher. The Chao1 and ACE indices showed significant differences between Kawasaki disease (KD) patients and healthy controls (p < 0.001), indicating altered microbial richness in the KD group. In contrast, the Simpson and Fisher indices did not show significant differences, suggesting that microbial evenness remains relatively unchanged. Boxplots display the distribution of diversity indices across the two groups, with red representing controls and blue representing KD patients.

**Supplementary Figure 2. Beta Diversity Analysis Between Kawasaki Disease and Control Groups.** Beta diversity was evaluated using multiple metrics, including the Bray-Curtis Index and Jensen-Shannon Divergence, with significance assessed through PERMANOVA (p = 0.001) and ANOISM (p = 0.001). The analyses showed significant differences in bacterial community composition between Kawasaki disease (KD) patients and healthy controls across all metrics. Each plot depicts the clustering of samples based on the respective beta diversity metric, with red points representing controls and blue points representing KD patients. The distinct separation between the two groups highlights substantial shifts in microbial composition associated with KD.

**Supplementary Figure 3. Key Gut Bacteria and Predictive Models for Kawasaki Disease.** (A) Relative abundances of *Bifidobacterium longum* and *Bifidobacterium bifidum* in control and Kawasaki disease (KD) groups. (B) The *Eggerthella lenta*/*Bacteroides ovatus* ratio was significantly elevated in KD patients compared to controls (p < 0.001). (C) Receiver operating characteristic (ROC) curve showing the predictive performance of the *Eggerthella lenta*/*Bacteroides ovatus* ratio for KD, with an area under the curve (AUC) of 0.859, a cutoff value of 0.018, and an odds ratio of 29.922 (95% CI: 10.697–83.700, p < 0.001). (D) Relative abundance of *Eggerthella lenta* in KD and control groups, showing significantly higher levels in the KD group (p < 0.001). (E) ROC curve illustrating the predictive value of *Eggerthella lenta* to age ratio for KD, with an AUC of 0.845, a cutoff value of 0.012, and an odds ratio of 20.114 (95% CI: 7.504–53.918, p < 0.001). Error bars in panels (A), (B), and (D) represent standard deviations.

**Supplementary Figure 4. Correlation Analysis of Inflammatory Markers, Gut Microbial Ratios, and a specific metabolic pathway in Kawasaki Disease.** Each scatter plot included a linear regression line to illustrate the direction and strength of the correlations. (A) Correlation between C-reactive protein (CRP) levels and blood cell types in Kawasaki disease (KD). (B) Relationship between CRP levels and the *Eggerthella lenta*/*Bacteroides ovatus* ratio, showing a significant negative correlation (r = -0.295, p = 0.025). (C) Correlation between the *Eggerthella lenta*/*Bacteroides ovatus* ratio and the flavone and flavonol biosynthesis pathway, demonstrating a strong negative association (r = -0.465, p < 0.001).
