# Supplementary figures and images for: Eggerthella lenta down regulated flavone and flavonol biosynthesis promoted Kawasaki disease
Source: Virulence. 2025 May 31;16(1):2512401. doi: 10.1080/21505594.2025.2512401 (PMC12128670; doi:10.1080/21505594.2025.2512401)

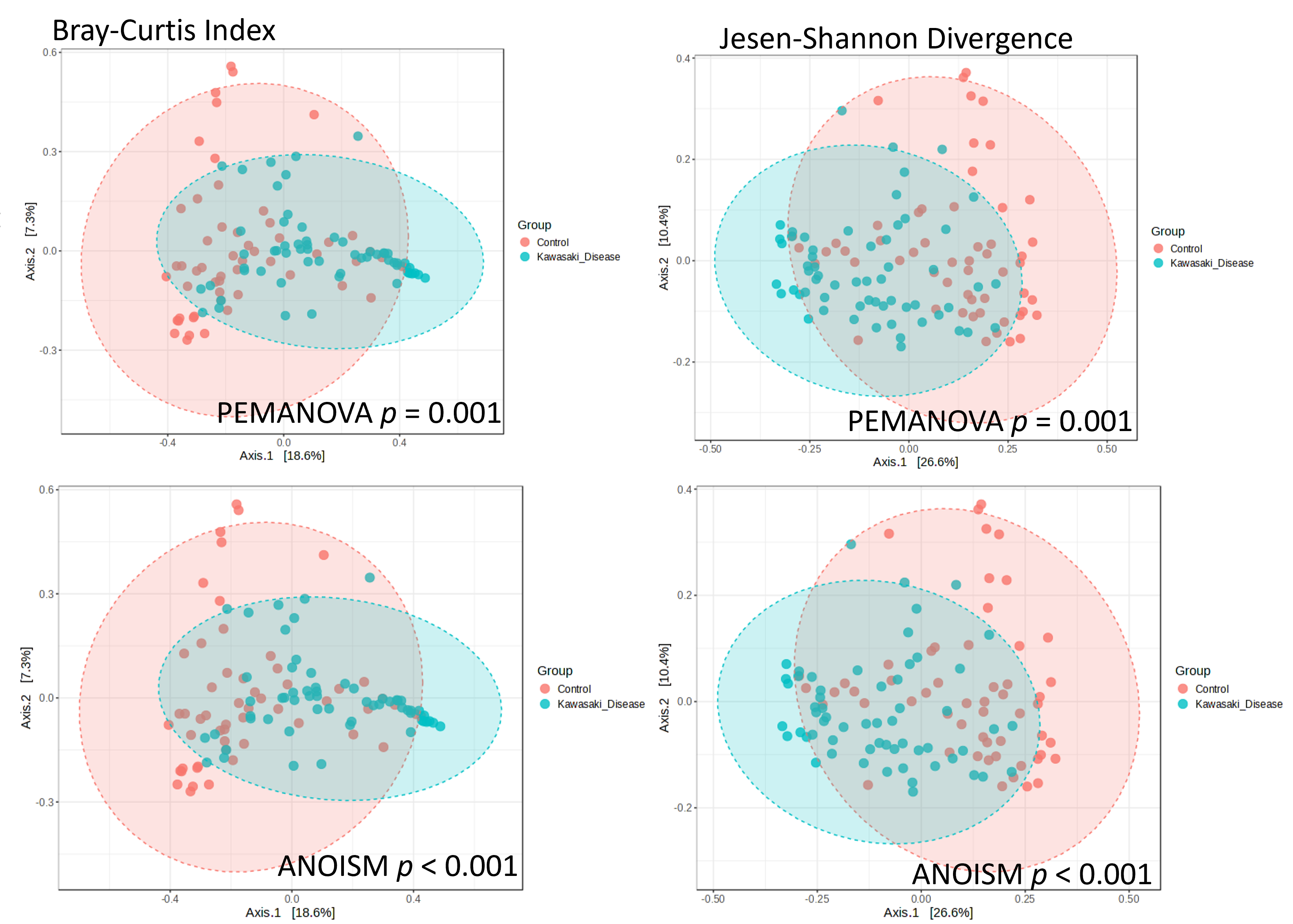

Supplement: Supplementary Figure 2.tif [file KVIR_A_2512401_SM6172.tif]
